# Endovascular Repair of Aortobronchial Fistula after Bentall Procedure

**DOI:** 10.1155/2018/1975756

**Published:** 2018-04-03

**Authors:** Dane D. Gruenebaum, Ray Graf, Thomas Alexander, Sergio Tavares, Salim Surani

**Affiliations:** ^1^Division of Cardiology, Corpus Christi Medical Center, Corpus Christi, TX 78412, USA; ^2^Department of Surgery, Corpus Christi Medical Center, Corpus Christi, TX 78412, USA; ^3^Division of Pulmonary Medicine, Corpus Christi Medical Center, Corpus Christi, TX 78412, USA

## Abstract

Aortobronchial fistula (ABF) is a rare complication of aortic repair seen with both surgical and endovascular manipulation. The available literature largely refers to the need for surgical repair. We are not aware of any reports of endovascular repair after the Bentall procedure. This report describes a patient who after the Bentall procedure presented with massive hemoptysis from ABF, a multidisciplinary team decided on endovascular repair due to patient frailty to avoid redo sternotomy. We believe this is the first case report of endovascular repair and represents the success of multidisciplinary collaboration.

## 1. Introduction

Aortobronchial fistula (ABF) is a rare complication after endovascular or surgical manipulation. Available literature has suggested that thoracic endovascular aortic repair (TEVAR) may be an option for selected patients [1]; however, the data have been limited to case reports and series, none of which to our knowledge were in patients after the Bentall procedure. Therapy for these patients previously has been restricted to redo sternotomy and aortic repair with high morbidity and mortality. The complex aortic disease seen in a patient with prior aortic graft and degeneration with fistula formation represents a significant number of unknowns compared to TEVAR in native aorta for which there is significant experience, such as whether it would be technically feasible, without the need for adjunctive anchoring for endoseal. Long-term durability is also unknown, but the desire to avoid the high morbidity and mortality of emergent redo sternotomy in a frail patient drove the team. We hereby present such a case.

## 2. Case Report

**Figure 1:**
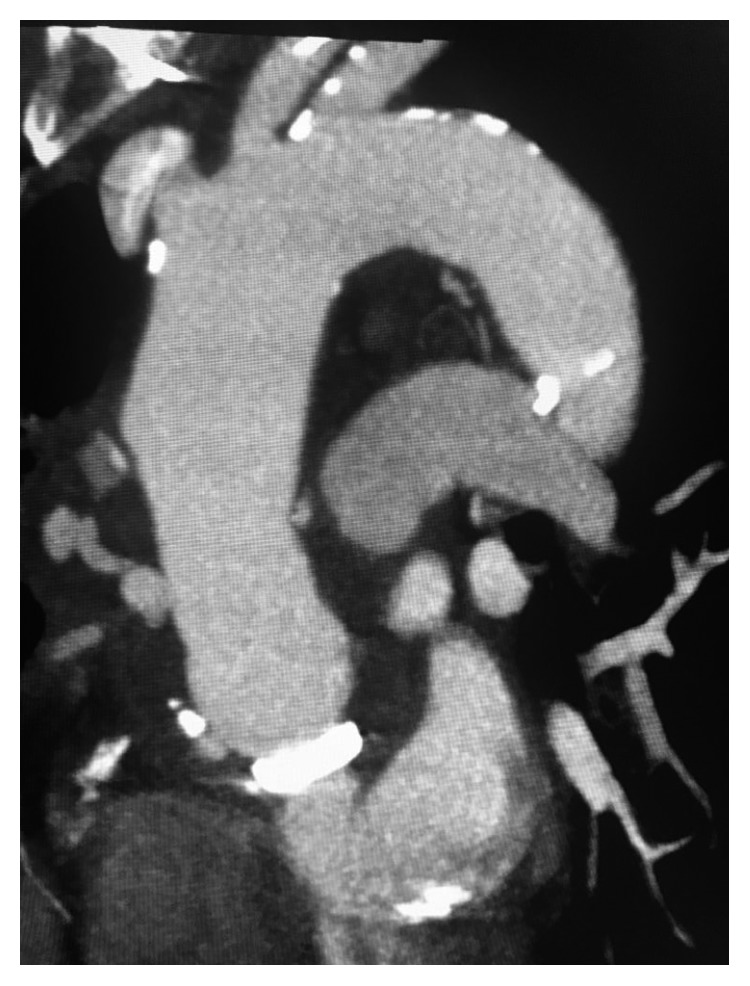
CTA showing pseudoaneurysmal degeneration of the aortic graft with fistulous flow to the right upper lobe.

A 66-year-old Caucasian female presented to an outlying emergency department (ED) for one-hour history of massive hemoptysis. Computed tomographic angiography (CTA) was performed demonstrating pseudoaneurysmal degeneration of the aortic graft and fistulous flow to the right upper lobe (RUL) bronchus (Figure 1).

She had a known history of Bentall repair with mechanical aortic valve replacement (AVR) on warfarin for approximately 17 months prior, with concurrent coronary artery bypass graft (CABG) × 1. Her past medical history was otherwise significant for noncompliance, non-oxygen-dependent chronic obstructive pulmonary disease (COPD), paroxysmal atrial fibrillation, current tobacco abuse, hypothyroidism, major depressive disorder, and past history of intravenous drug abuse. Other surgical history includes lumbar laminectomy and hysterectomy. Her home medications included tiotropium, warfarin, amlodipine, olmesartan, carvedilol, simvastatin, levothyroxine, fluticasone/salmeterol, and escitalopram.

The INR (1.72) was reversed with administration of vitamin K and fresh frozen plasma (FFP) at the outlying ED, and she was transferred for cardiothoracic (CT) surgical evaluation. Surgery promptly involved interventional cardiology (IC) and pulmonary/critical care medicine (PCCM). Bronchosocpy revealed concordant right upper lobe (RUL) hemorrhage for which epinephrine was unable to cessate the bleeding.

**Figure 2:**
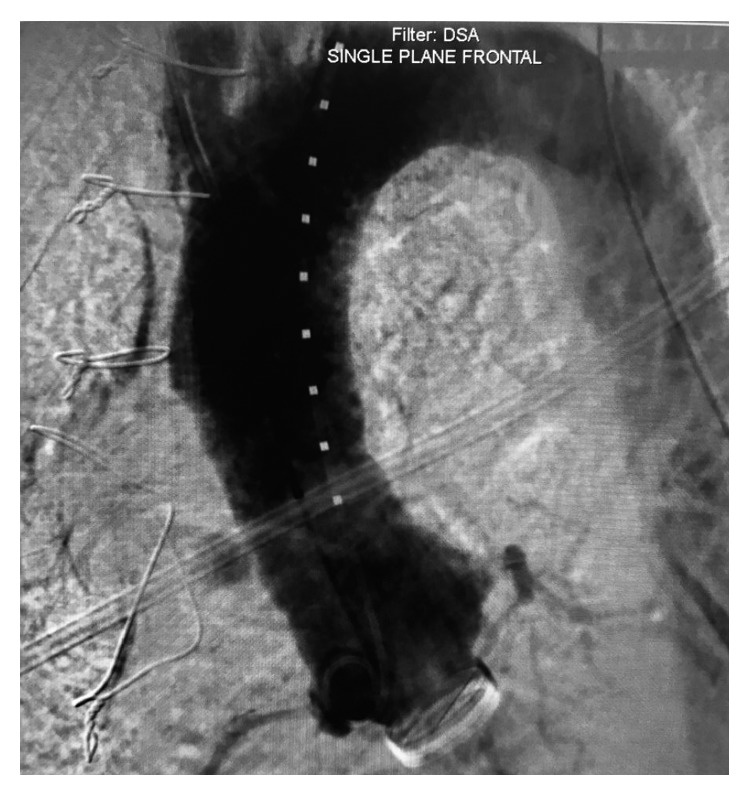
Aortic root angiogram with marker pigtail to verify sizing.

With ongoing blood loss and hemoglobin drop from 13.5 g/dl to 10.4 g/dl and the desire to avoid redo sternotomy, the decision to attempt multidisciplinary TEVAR was selected. After administration of general anesthesia, CT surgery performed exposure of the left subclavian artery, subsequently interventional cardiology cannulated the left subclavian artery with the modified Seldinger technique and a 6 Fr sheath was placed with advancement of a marker pigtail catheter and angiographic assessment for landing zones (Figure 2).

The sheath was subsequently upsized over an Amplatz superstiff wire for an 18 Fr sheath. Based on CTA and invasive angiographic findings, a 36 mm diameter × 45 mm length Gore aortic extension cuff was selected. It was deployed satisfactorily, and then, a Gore Q50 compliant aortic occlusion balloon was used for postdilatation.

**Figure 3:**
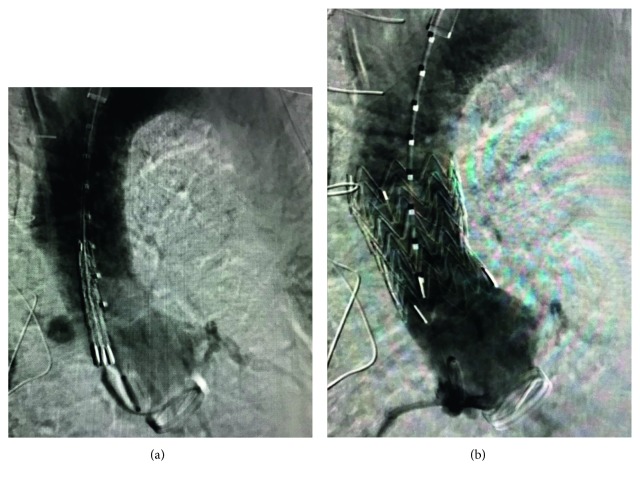
Aortic root angiogram showing placement of graft and postdeployment with cessation of fistulous flow.

Repeat angiographic assessment revealed cessation of flow into the pseudoaneurysmal/fistulous segment (Figure 3). Subsequent CTA again showed cessation of flow. Given concern for the respiratory flora colonization of the now atretic fistulous tract, the patient was placed on empiric IV antibiotic therapy for six weeks. The patient did well and was discharged home.

## 3. Discussion

Aortobronchial fistula has been previously described as a rare cause of hemoptysis after prior aortic operative/endovascular manipulation [2]. Historically, this was thought to only be correctable operatively otherwise felt to be uniformly fatal [3].

Even with operative intervention, there was significantly high operative morality for the emergent redo sternotomy, where the retrospective series reported 20% [4].

TEVAR for ABF has been around since 1996 [5] More recent limited case series have suggested that TEVAR may be a viable option for ABF [6], in fact, one series by Jonker et al. suggested it may be the preferred strategy for ABF, with low operative mortality (3%) and low need for operative intervention in the first 30 days after TEVAR (3%) [7].

Colonization and infection of the endograft via the ABF of respiratory flora is a concern, where staged repair and pulmonary resection may be an option to minimize this risk if TEVAR is to be considered. [8].

With the limited frequency of ABF, which has not been well defined, there are no guidelines for the use of TEVAR versus conventional operative repair. Similarly, there does not appear to be a consensus on what may be appropriate antibiotic therapy for these individuals, though some have advocated a regimen similar to uncomplicated endocarditis, six weeks of broad-spectrum parenteral antibiotics followed by lifelong oral suppressant therapy [7]. For frankly infected grafts, surgical explantation with in situ reconstruction, or extra-anatomic bypass for high-risk patients has been shown to be feasible [8].

Many factors drove the decision to attempt TEVAR for our patients with ABF; first, we were fortunate enough to have a relatively stable presentation where we had time to take a multidisciplinary approach. The surgeon who performed her Bentall/CABG wanted to avoid the morbidity and mortality of emergent redo sternotomy. Finally, the patient relative frailty and the favorable anatomic position of the ABF (3 cm above the sinotubular junction and 4 cm below the brachiocephalic artery) undoubtedly made it feasible.

## 4. Conclusion

We believe that this is the first case report of TEVAR for ABF after Bentall. Caution should be taken in selecting an appropriate patient anatomy, factoring in comfort of the operators including multidisciplinary team evaluation depending on endovascular expertise and device as well as operative comfort for a rescue if emergent operative conversion becomes necessary.
